# A859L Plays a Critical Role in Facilitating Efficient African Swine Fever Virus Replication

**DOI:** 10.3390/v18101059

**Published:** 2026-09-24

**Authors:** Xiangtao Zhu, Hongyu Yan, Wanhui Zhou, Haiyu Su, Xiumei Huang, Chongxian Deng, Yuxing Li, Yali Wang, Yanyan Chang, Wenping Yang, Haixue Zheng

**Affiliations:** 1 School of Biological and Pharmaceutical Engineering, Lanzhou Jiaotong University, Lanzhou 730070, China; 15236287125@163.com (X.Z.);; 2 Key Laboratory of Microbial Resources Exploitation and Application of Gansu Province, Lanzhou 730070, China; 3 State Key Laboratory of Animal Disease Control and Prevention, Lanzhou Veterinary Research Institute, Chinese Academy of Agricultural Sciences, Lanzhou 730046, China

**Keywords:** African swine fever virus, A859L, RNA helicase, virus replication

## Abstract

African swine fever virus (ASFV) is a highly pathogenic agent that poses a severe threat to the global swine industry. The biological function of A859L, an ASFV-encoded RNA helicase, remains largely elusive. Here, we show that A859L is highly conserved across diverse ASFV isolates and belongs to the SF2 superfamily of RNA helicases, possessing characteristic Walker A (GKT) and Walker B (DECH) motifs. Transcriptomic profiling at multiple time points post-infection further revealed that *A859L* is a late-expressed gene. Functional assays demonstrated that siRNA-mediated knockdown of *A859L* significantly inhibited progeny virus production in both susceptible macrophages (PAMs and BMDMs) and WSL cells. This knockdown also markedly reduced the expression of viral proteins p30 and p72, as well as the intracellular mRNA levels of other viral helicase genes (*QP509L*, *Q706L*, *D1133L*, and *B962L*), underscoring the vital role of A859L in the viral life cycle. Conversely, A859L overexpression enhanced viral propagation. Notably, pharmacological inhibition of viral helicase activity effectively suppressed ASFV replication. Collectively, our data establish A859L as a critical determinant of ASFV replication and a promising candidate for antiviral development.

## 1. Introduction

African swine fever (ASF), caused by the African swine fever virus (ASFV), represents a critical global threat to the swine industry [1,2], causing acute hemorrhagic fever with mortality rates reaching up to 100% in domestic pigs [3]. First isolated in Kenya in 1921, ASFV has since spread to numerous countries and regions worldwide [4,5,6]. The World Organisation for Animal Health (WOAH) lists ASF as a notifiable animal disease [7]. Since its emergence in China in August 2018 [8], ASF has caused billions of dollars in losses annually [1] and is categorized as a Category I animal infectious disease in China [9]. ASFV is a large double-stranded DNA (dsDNA) virus of the genus *Asfarvirus* within the *Asfarviridae* family [10]. Its genome ranges from 170 to 194 kb [11] and contains 151 to 167 open reading frames (ORFs) encoding at least 160 proteins [12], most of which remain functionally uncharacterized. Currently, there are no safe and effective vaccines or approved antiviral drugs available [13], making the prevention and control situation extremely grim [14].

Viral RNA helicases serve as essential motor proteins that unwind double-stranded RNA (dsRNA) or dissociate RNA-protein complexes [15,16], driving critical processes throughout the viral life cycle, including genome replication, transcription, and translation [17,18]. The majority of these enzymes are classified into helicase superfamily 1 (SF1) or superfamily 2 (SF2), both of which share conserved catalytic core domains [19]. Structurally, active RNA helicases typically comprise N- and C-terminal domains that flank the core to form an RNA substrate channel and an active site pocket accommodating nucleotide triphosphates (NTPs) [20], divalent metal ions [21], and catalytic water molecules [22]. Notably, SF2 helicases are defined by conserved Walker A (GxGKT/S) and Walker B (DExD/H) motifs that are strictly required for catalytic activity [23,24]. Owing to their high conservation among diverse viruses and the lack of host homologs, viral RNA helicases constitute promising targets for broad-spectrum antiviral drug development.

ASFV encodes five putative RNA helicases: D1133L, B962L, QP509L, Q706L, and A859L [25]. Although the SF2 helicases QP509L and Q706L have been shown to co-localize at viral factories and are essential for viral propagation [25], the functional relevance of the remaining candidates is poorly understood. Notably, a recombinant ASFV with a deletion of the *A859L* gene replicated as efficiently as the parental strain in both swine macrophage cultures and domestic pigs, indicating that *A859L* is genetically dispensable under standard conditions [26]. This apparent non-essentiality raises important questions regarding the actual contributions of these uncharacterized helicases to the viral life cycle.

To address this gap, we systematically characterized the biological properties of A859L. Our analysis reveals that A859L is highly conserved across diverse ASFV lineages. Contrary to the genetic dispensability of *A859L*, functional assays combining siRNA-mediated knockdown and ectopic overexpression demonstrate that the A859L protein is critically required for efficient ASFV replication. Structurally, we resolved the spatial arrangement of the Walker A and B motifs within the A859L core domain, confirming its canonical helicase architecture. Most importantly, we show that pharmacological inhibition of A859L helicase activity profoundly suppresses viral replication, highlighting A859L as a viable target for antiviral drug development.

## 2. Materials and Methods

### 2.1. Cells

Primary porcine alveolar macrophages (PAMs) were isolated from bronchoalveolar lavage fluid, and porcine bone marrow-derived macrophages (BMDMs) were generated from bone marrow harvested from the femoral and tibial medullary cavities of 30-day-old specific-pathogen-free (SPF) pigs, as previously described [27]. To support efficient expansion and differentiation of BMDMs, recombinant porcine granulocyte-macrophage colony-stimulating factor (pGM-CSF; 711-PG; R&D Systems, Minneapolis, MN, USA) was added to RPMI 1640 medium (C22400500BT, Gibco, Thermo Fisher Scientific, Waltham, MA, USA) at a final concentration of 10 ng/mL. Both PAMs and BMDMs were cultured in RPMI 1640 supplemented with 10% fetal bovine serum (FBS; A5256701, Gibco, Thermo Fisher Scientific, Waltham, MA, USA) and 100 U/mL penicillin–streptomycin, in a humidified incubator with 5% CO_2_ at 37 °C. The porcine kidney epithelial cell line PK-15 (CCL-33) purchased from ATCC and the wild boar lung cell line WSL generously provided by Prof. Haixue Zheng were maintained in DMEM (C11995500BT, Gibco, Thermo Fisher Scientific, Waltham, MA, USA) supplemented with 10% FBS and 100 U/mL penicillin–streptomycin under the same culture conditions.

### 2.2. Viruses

The ASFV strain CN/GS/2018 was provided by and is maintained at the Lanzhou Veterinary Research Institute, Chinese Academy of Agricultural Sciences (Lanzhou, China). The virus was propagated and titrated in BMDMs. All experiments involving ASFV were conducted in Biosafety Level 3 (BSL-3) containment facilities at the Lanzhou Veterinary Research Institute, in strict compliance with institutional biosafety protocols.

### 2.3. Antibodies and Reagents

Monoclonal mouse anti-Streptavidin tag antibody (A01732) was purchased from GenScript (Nanjing, China). Monoclonal mouse antibodies against β-actin (66009-1-Ig) and GFP tag (66002-1-Ig) were obtained from Proteintech (Wuhan, China). Monoclonal mouse anti-ASFV p30 and polyclonal rabbit anti-ASFV p72 antibodies were generated in-house. Horseradish peroxidase (HRP)-conjugated AffiniPure goat anti-mouse IgG (H + L) (SA00001-1) and goat anti-rabbit IgG (H + L) (SA00001-2) secondary antibodies were sourced from Proteintech. Fluorescently labeled secondary antibodies-Alexa Fluor 488 goat anti-mouse IgG (H + L) (A-11001), Alexa Fluor 488 goat anti-rabbit IgG (H + L) (A-11008), Alexa Fluor 568 goat anti-mouse IgG (H + L) (A-11004), and Alexa Fluor 568 goat anti-rabbit IgG (H + L) (A-11036) were acquired from Invitrogen (Thermo Fisher Scientific, Waltham, MA, USA). DAPI (C1002) was purchased from Beyotime Biotechnology (Shanghai, China).

### 2.4. Plasmid Construction and DNA Transfection

To generate the eukaryotic expression plasmid pRK-A859L-Strep, the coding sequence of *A859L* was amplified from ASFV (strain CN/GS/2018) genomic DNA using 2 × Phanta Max Master Mix (Dye Plus; P525, Vazyme, Nanjing, China) and subsequently cloned into the Hind *III*/BamH *I*-linearized pRK-Strep vector via seamless cloning (Vazyme, C112). Additionally, a Strep-tagged, siRNA-resistant A859L expression plasmid, designated pRK-srA859L-Strep, was constructed by introducing silent mutations into the siRNA-targeting region without altering the encoded amino acid sequence. Plasmid DNA was transfected into PK-15 and WSL cells using Lipofectamine™ 3000 (L3000015, Invitrogen, Thermo Fisher Scientific, Waltham, MA, USA), following the manufacturer’s instructions, and cells were incubated for 24 h post-transfection.

### 2.5. Hemadsorption Assay

The ASFV strain CN/GS/2018 was quantified using a hemadsorption assay as previously described, with minor modifications [28]. Briefly, BMDMs (5 × 10^4^ cells per well) were seeded into 96-well plates and infected with serial 10-fold dilutions of ASFV. Following incubation at 37 °C for 48 h, porcine red blood cells (approximately 5 × 10^5^ cells) resuspended in complete RPMI 1640 medium (supplemented with 10% FBS and 100 U/mL penicillin–streptomycin) were added to each well. Hemadsorption was monitored daily by light microscopy for up to 5 days. The 50% hemadsorption dose (HAD_50_) was calculated using the Reed–Muench method [29].

### 2.6. RNA Extraction and RT-qPCR

BMDMs or PAMs were seeded in 12-well plates and infected with ASFV at a multiplicity of infection (MOI) of 0.1 or 0.5, respectively. At the indicated time points post-infection, total RNA was extracted using RNAiso Plus reagent (9109, Takara, Dalian, China) following the manufacturer’s instructions. First-strand cDNA was synthesized from 1 µg of total RNA using the HiScript II 1st Strand cDNA Synthesis Kit (+gDNA wiper; Vazyme, China, R212-01). Quantitative real-time PCR (qPCR) was performed on a Bio-Rad CFX Real-Time PCR Detection System (Bio-Rad, Hercules, CA, USA) using ChamQ Universal SYBR qPCR Master Mix (Q711-02, Vazyme, Nanjing, China). Primer sequences used for RT-qPCR are listed in Table 1.

### 2.7. Western Blot Analysis

Cells were washed once with PBS (pH 7.4) and lysed in SDS lysis buffer (20 mM Tris–HCl, pH 7.5, 150 mM NaCl, 5 mM EDTA, 1% SDS, and 10% glycerol). Cell lysates were denatured by boiling at 95 °C for 10 min, resolved on a 12% SDS–PAGE gel, and electrotransferred onto nitrocellulose (NC) membranes (Millipore, Darmstadt, Germany). Membranes were blocked with 5% (*w*/*v*) non-fat milk in Tris-buffered saline (TBS) for 1 h at room temperature, followed by incubation with primary antibodies diluted in blocking buffer overnight at 4 °C. After thorough washing with TBS containing 0.1% Tween-20 (TBST), membranes were probed with horseradish peroxidase (HRP)-conjugated secondary antibodies for 1 h at room temperature. Protein bands were detected using an enhanced chemiluminescence (ECL) substrate (Millipore) and imaged on a ChemiDoc™ MP Imaging System (Bio-Rad, USA).

### 2.8. Immunofluorescence Assays (IFA) and Confocal Microscopy

Cells transfected with pRK-A859L-Strep were washed with PBS, fixed with 4% paraformaldehyde (PFA), permeabilized with 0.2% Triton X-100, and blocked with 5% bovine serum albumin (BSA). The cells were then incubated with the indicated primary antibodies overnight at 4 °C. After thorough washing, the cells were incubated with fluorescently labeled secondary antibodies for 1 h at room temperature. Nuclei were counterstained with DAPI. Fluorescence images were acquired using a Zeiss LSM980 (Carl Zeiss, Jena, Germany) confocal laser scanning microscope with a 63× oil immersion objective.

### 2.9. Gene Silencing Using siRNA

Chemically synthesized 21-nucleotide siRNA duplexes targeting *A859L* mRNA were obtained from Tsingke Biotech (Beijing, China). PAMs or BMDMs were seeded in 12-well plates and transfected with 50 nM of either a non-targeting control siRNA (siCtrl) or *A859L*-specific siRNA (si*A859L*) using Lipofectamine™ RNAiMAX Reagent (13778150, Invitrogen, Thermo Fisher Scientific, Waltham, MA, USA) according to the manufacturer’s instructions. Knockdown efficiency was assessed by qPCR using gene-specific, predesigned primers to measure *A859L* transcript levels at the indicated time points. The siRNA sequences are listed in Table 2.

### 2.10. Structure Visualization of the Helicase Active Site in A859L

The amino acid sequence of A859L was submitted to the AlphaFold 3 (AF3) web server. Three-dimensional structural models were generated using default AF3 parameters, which included the prediction of backbone and side-chain conformations along with per-residue confidence scores (pLDDT). The resulting structures were visualized using PyMOL (version 2.5.5).

### 2.11. Cell Viability Assay

Cells were seeded in 96-well plates 16 h prior to treatment and subsequently exposed to different drug concentrations for 36 h. After treatment, 10 μL of Cell Counting Kit-8 (CCK-8; Abbkine, Wuhan, China, BMU106) solution was added to each well, followed by incubation at 37 °C for 2 h. The optical density (OD) at 450 nm was measured using a microplate spectrophotometer, and cell viability was calculated according to the manufacturer’s instructions.

### 2.12. Statistical Analysis

All data are presented as the mean ± standard deviation (SD) from three independent experiments. For comparisons between two groups, Student’s t-test was used. For comparisons involving three or more groups, one-way analysis of variance (ANOVA) was performed using GraphPad Prism software (version 8.4.3). Statistical significance was defined as *p* < 0.05. In the figures, significance is denoted as follows: * *p* < 0.05, ** *p* < 0.01, *** *p* < 0.001, and not significant (ns): *p* > 0.05.

## 3. Results

### 3.1. A859L Is Highly Conserved Across Diverse ASFV Isolates

The ORF of the *A859L* gene in the ASFV CN/GS/2018 genome lies between the *A238L* and *A179L* genes and encodes an 858-amino-acid protein, A859L. To evaluate sequence conservation across the genetic and geographic diversity of ASFV—and, in particular, to ensure representation of multiple p72 (*B646L*)-based genotypes—we performed a multiple sequence alignment of the full-length A859L amino acid sequences from 25 representative isolates retrieved from the NCBI Virus database, using compositionally adjusted substitution matrices. We chose to span genotype I (e.g., BA71V) and genotype II (e.g., Georgia_2008/1 together with the Eurasian field strains, including Pig/HLJ/2018), as well as several African isolates (from Nigeria, Mozambique, Tanzania, Malawi, and South Africa); geographically, it covers Asia, Europe, and Africa. Because the full-length alignment was identical at all but a few positions, Figure 1 displays, for clarity, only the windows that contain amino acid substitutions; every position outside these windows was 100% conserved across all 25 isolates. Over the entire protein, the amino acid sequence identity was approximately 99%, and the substitutions that did occur were restricted to a limited number of discrete sites (e.g., positions 109, 145, 381, 382, 421, 426, 751, and 829) and were detected predominantly in only a small subset of strains—notably BA71V and Pig/Henan/123014/2022—whereas the majority of isolates, including representatives of each genotype, matched the reference sequence throughout (Figure 1). Collectively, these data demonstrate that A859L is highly conserved across ASFV isolates of different genotypes and geographic origins.

### 3.2. Structural Characterization of the Helicase Active Site in A859L

It was reported that A859L is an RNA helicase of ASFV [25,26]. To elucidate the spatial structure of A859L, its amino acid sequence was submitted to the AlphaFold 3 (AF3) prediction pipeline. The predicted structure was visualized using PyMOL (version 2.5.5). Our analysis revealed that the core domain of A859L adopts a canonical conformation of an RNA helicase active site pocket (Figure 2A). To further verify the presence of a helicase domain, we analyzed the A859L sequence using the InterProScan (version 5.78) web server. The results confirmed that A859L harbors the conserved motifs characteristic of SF2 superfamily helicases, specifically Walker A (^195^GKT^197^) and Walker B (^297^DECH^300^), suggesting its involvement in ATP hydrolysis. Notably, both motifs are positioned within the core catalytic pocket (Figure 2B). Furthermore, structural mapping indicates that the motif sites in A859L are clustered in close proximity, strongly suggesting that this region constitutes the functional helicase activity pocket (Figure 2C).

### 3.3. Transcriptional Kinetics and Intracellular Localization of A859L

To characterize the transcriptional kinetics of *A859L*, porcine alveolar macrophages (PAMs) were infected with ASFV and harvested at 0, 3, 6, 9, 12, 18, 24, 36, and 48 h post-infection (hpi). Total RNA was extracted, and *A859L* transcript levels were quantified by RT-qPCR. The expression levels of *CP204L* (encoding the viral p30 protein), a well-established early-gene marker, and *B646L* (encoding the viral p72 protein), a canonical late-gene marker, were analyzed in parallel for temporal comparison. The intracellular viral *CP204L* mRNA levels increased rapidly as early as 3 hpi, whereas *A859L* transcripts showed a sharp upregulation beginning at 9 hpi. Notably, *A859L* expression was undetectable until 9 hpi, consistent with its classification as a late-transcribed gene (Figure 3A). A similar expression profile was observed in ASFV-infected porcine bone marrow–derived macrophages (BMDMs; Figure 3B). Collectively, these findings confirm that *A859L* is transcribed during the late phase of the ASFV replication cycle. To assess the subcellular localization of A859L, PK-15 cells were transfected with either an empty vector or the pRK-A859L-Strep plasmid expressing A859L-Strep protein, followed by an immunofluorescence assay (IFA). The IFA results demonstrated that the A859L protein is predominantly distributed in the cytoplasm (Figure 3C).

### 3.4. A859L Is Crucial for ASFV Replication

To investigate the role of A859L in the ASFV life cycle, PAMs were transfected with small interfering RNAs (siRNAs) targeting *A859L*, and efficient knockdown of *A859L* mRNA was confirmed by RT-qPCR. ASFV encodes several putative RNA helicases in addition to A859L, including QP509L, Q706L, D1133L, and B962L. To determine whether *A859L* depletion triggers compensatory upregulation of these helicases or selectively affects their expression, we quantified their mRNA levels by RT-qPCR. The mRNA levels of *QP509L*, *Q706L*, *D1133L*, and *B962L* were all significantly reduced in ASFV-infected *A859L*-knockdown PAMs, mirroring the reduction observed for viral genes *CP204L* and *B646L* (Figure 4A). This parallel decrease may indicate that A859L does not specifically regulate the transcription of other viral helicases; rather, its depletion leads to a broad, non-selective attenuation of viral gene expression, consistent with a role for A859L as a general facilitator of viral RNA metabolism within the replication–transcription complex. At the protein level, Western blot analysis revealed a marked decrease in the intracellular levels of p72 and p30 in ASFV-infected *A859L*-knockdown PAMs compared with control cells (Figure 4B). Furthermore, hemadsorption assays demonstrated that silencing of *A859L* substantially impaired the production of extracellular infectious virions (Figure 4C).

To validate these findings in an additional ASFV-susceptible mononuclear phagocyte population, BMDMs were similarly transfected with *A859L*-targeting siRNAs. Quantitative RT-qPCR analysis showed that knockdown of *A859L* in ASFV-infected BMDMs resulted in a significant reduction in the viral mRNA levels of *CP204L*, *B646L*, and the other ASFV RNA helicases (Figure 4D). Consistent with the results obtained in PAMs, Western blot analysis (Figure 4E) and hemadsorption assays (Figure 4F) confirmed markedly impaired viral protein expression and infectious virion production, respectively.

Collectively, these results demonstrate that A859L is important for efficient ASFV replication across multiple susceptible macrophage types and that its function is not restricted to the regulation of individual viral helicases but rather supports global viral gene expression.

### 3.5. A859L Promotes ASFV Replication

We next investigated whether A859L is important for efficient ASFV replication in additional target cell types. In addition to domestic pigs, wild boars are also susceptible to ASFV [30]. Therefore, we assessed the impact of A859L on ASFV replication in WSL cells, a wild boar lung cell line. Quantitative RT-qPCR analysis demonstrated that efficient knockdown of *A859L* in ASFV-infected WSL cells significantly reduced the intracellular mRNA expression of viral genes *CP204L* and *B646L*, as well as other viral helicase genes, including *QP509L*, *Q706L*, *D1133L*, and *B962L* (Figure 5A). Furthermore, Western blot analysis revealed a marked downregulation of viral proteins p72 and p30 in ASFV-infected *A859L*-knockdown WSL cells (Figure 5B). Hemadsorption assays further demonstrated that *A859L* knockdown substantially impaired the production of extracellular infectious virions (Figure 5C). These results are consistent with the role of A859L observed in porcine macrophage cell types, including PAMs and BMDMs (Figure 4).

To further evaluate the role of A859L in ASFV replication, WSL cells were transfected with either an empty vector or a Strep-tagged A859L expression plasmid for 18 h and subsequently infected with ASFV. Western blot analysis revealed that A859L overexpression significantly increased the intracellular levels of viral proteins p72 and p30 in a dose-dependent manner (Figure 5D). Consistently, RT-qPCR analysis showed elevated intracellular mRNA levels of the viral genes *CP204L* and *B646L* (Figure 5E), and hemadsorption assays demonstrated a marked increase in the production of extracellular infectious virions upon A859L overexpression (Figure 5F). Together, these results indicate that A859L facilitates ASFV replication.

To verify the functional specificity of A859L in viral replication, we performed a knockdown–rescue experiment in WSL cells. Cells were transfected with either control siRNA (siCtrl) or one of two *A859L*-specific siRNAs for 18 h, infected with ASFV for 12 h, and subsequently transfected with a plasmid expressing a siRNA-resistant, Strep-tagged A859L (pRK-srA859L-Strep). Western blot analysis demonstrated that siRNA-mediated knockdown of *A859L* significantly reduced intracellular levels of p72 and p30 compared with the siCtrl group, whereas re-expression of srA859L markedly restored these protein levels (Figure 5G). Quantitative RT-qPCR and hemadsorption assays further confirmed that both intracellular mRNA levels of viral genes *CP204L* and *B646L* (Figure 5H) and extracellular infectious virus production (Figure 5I) were significantly recovered upon srA859L re-expression. Although the rescue was partial rather than complete, this is likely attributable to differences in expression stoichiometry or post-translational kinetics between endogenous A859L and the exogenously expressed construct—a commonly observed limitation in transient rescue systems. Collectively, these findings confirm that A859L specifically promotes ASFV replication.

### 3.6. Helicase Activity Is Critical for ASFV Replication

Given that viral helicase activity plays vital roles in genome replication, transcription, and repair [31,32], we next investigated its importance for ASFV replication. Pritelivir, a potent antiviral compound known to inhibit the herpes simplex virus type 1 (HSV-1) UL5 helicase, was evaluated for its efficacy against ASFV. UL5 shares conserved ATPase motifs (Walker A/B) [33] with ASFV A859L and serves as the direct binding target of Pritelivir, which suppresses viral replication [34,35,36]. PAMs and BMDMs were infected with ASFV for 2 h and subsequently treated with varying concentrations of Pritelivir for an additional 36 h. Western blot analysis revealed that Pritelivir reduced the intracellular levels of viral p30 and p72 proteins in a concentration-dependent manner in both cell types (Figure 6A,B). Consistently, quantitative RT-qPCR analysis demonstrated that the intracellular mRNA levels of *CP204L*, *B646L*, and other ASFV RNA helicase genes *A859L*, *QP509L*, *Q706L*, *D1133L*, and *B962L* were significantly decreased following Pritelivir treatment in both PAMs (Figure 6C) and BMDMs (Figure 6D). Furthermore, hemadsorption assays showed that Pritelivir treatment markedly reduced the production of extracellular infectious virions (Figure 6E,F). Importantly, these antiviral effects were not attributable to cytotoxicity, as CCK-8 assays confirmed that cell viability remained unaffected at all tested concentrations (Figure 6G).

To rule out potential confounding effects on the transcription of ASFV RNA helicase genes, PAMs and BMDMs were infected with ASFV for 2 h prior to Pritelivir treatment, and RT-qPCR was performed at 9 hpi, a time point preceding substantial viral DNA replication (Figure 3). The results indicated that the intracellular mRNA levels of *CP204L*, *B646L*, and other ASFV RNA helicase genes remained unchanged upon inhibitor treatment in both PAMs (Figure S2A) and BMDMs (Figure S2B). These data confirm that the observed reduction in viral replication resulted from impaired helicase activity rather than diminished expression of ASFV RNA helicases. Collectively, these findings suggest that Pritelivir suppresses ASFV replication by inhibiting helicase activity.

## 4. Discussion

Among ASFV-encoded proteins, A859L exhibits remarkable conservation across isolates of diverse genotypes and geographic origins, with an amino acid identity of 99% (Figure 1). This extraordinary sequence stability implies that A859L is under strong purifying selection, suggesting a vital role in the viral life cycle that is preserved across lineages. Such high conservation is typically characteristic of proteins performing essential functions critical for viral survival and propagation, rendering them attractive targets for antiviral intervention due to their reduced propensity for rapid resistance development [37]. For instance, highly conserved enzymes such as the ASFV DNA polymerase pG1211R [38] and RNA polymerase subunits (pNP1450L [39], pC315 and pH359L [40]) are indispensable for viral nucleic acid synthesis. Consistent with this paradigm, our findings that siRNA-mediated knockdown of *A859L* impairs ASFV replication (Figure 4) and that pharmacological inhibition of A859L helicase activity suppresses viral propagation (Figure 6) indicate that A859L plays a crucial role in efficient ASFV replication.

However, an earlier study reported that A859L is dispensable for ASFV replication and pathogenesis: blood-derived swine macrophages infected with a recombinant ASFV (ASFV-G-Δ*A859L*) replicated as efficiently as the parental Georgia strain, and in vivo experiments showed that ASFV-G-Δ*A859L* caused clinical disease indistinguishable from that of the parental virus [26]. We propose that this apparent discrepancy with our findings arises from several key experimental differences rather than fundamental contradictions. First, cellular context differs: we employed porcine alveolar macrophages (PAMs), differentiated bone marrow-derived macrophages (BMDMs), and the wild boar lung cell line WSL—all physiologically relevant target cells—whereas Ramirez-Medina et al. used blood-derived monocyte-macrophages. Variations in host factor availability across these cell types may differentially influence viral dependency on A859L. Second, viral genetic backgrounds differ: our study utilized ASFV CN/GS/2018, while Ramirez-Medina et al. used ASFV Georgia; distinct isolates may exhibit varying replication requirements in specific cellular environments. Third, and most critically, the nature of genetic perturbation differs fundamentally: stable gene deletion involves multi-step homologous recombination and selection, during which compensatory mutations or adaptive changes may arise to offset the loss of *A859L*. In contrast, our approaches—siRNA-mediated knockdown and acute inhibition of A859L enzymatic activity by Pritelivir in cell-based assays—reduce protein levels or block function rapidly without allowing time for such compensatory evolution. This acute functional blockade likely triggers immediate replication arrest before compensatory mechanisms can be activated, thereby revealing an essential requirement that may be masked in adapted deletion mutants. Collectively, these considerations suggest that A859L is conditionally essential, with its importance modulated by cellular environment, viral genetic background, and the temporal dynamics of protein depletion.

As a large dsDNA virus, ASFV relies on A859L as a molecular engine driving efficient replication. We hypothesize that through its RNA helicase activity and dsRNA-binding capability, A859L resolves secondary structural obstacles in viral mRNA, thereby facilitating precise regulation of viral transcription and translation. An important question raised by our findings is whether A859L functions independently or cooperatively within the ASFV replication machinery. ASFV encodes multiple proteins involved in nucleic acid metabolism: DNA polymerase pG1211R localizes to viral factories and is essential for propagation [41]; RNA polymerase subunits pC315R and pH359L suppress host translation via PKR-eIF2α activation and dampen inflammatory responses [40]; and five putative RNA helicases (D1133L, B962L, QP509L, Q706L, and A859L) are encoded in the genome. Notably, in our early-infection transcriptomic dataset, *A859L* knockdown did not trigger compensatory upregulation of other helicase genes nor selectively alter expression of any single replication factor. Instead, we observed a modest global reduction in late viral gene expression—a pattern also recapitulated in WSL cells (Figure 5A–C)—consistent with a model in which A859L contributes to the overall efficiency of viral RNA metabolism, potentially by facilitating RNA unwinding during transcription or resolving mRNA secondary structures, rather than by directly regulating other helicases. Future studies employing co-immunoprecipitation, proximity-dependent biotinylation, or combinatorial knockdown approaches will be essential to map the physical and functional interactions of A859L within the viral replication–transcription complex and to elucidate the mechanistic basis of its conditional essentiality.

Viruses have evolved to encode RNA helicases that play pivotal roles in regulating viral genome expression and facilitating replication. For instance, the nonstructural protein 3 (NS3) of Zika virus (ZIKV), a member of the SF2 superfamily, possesses a canonical DExH RNA helicase domain that unwinds viral dsRNA to enable nascent strand synthesis; consequently, impairing NS3 helicase activity severely disrupts viral replication [42]. Consistent with this paradigm, our results identify A859L as a putative SF2-type RNA helicase characterized by a typical DExH-box domain containing specific Walker A (^195^GKT^197^) and Walker B (^297^DECH^300^) (Figure 2). The therapeutic potential of targeting A859L is further corroborated by our finding that Pritelivir potently suppresses ASFV replication in both PAMs and BMDMs. Pritelivir is a well-established inhibitor of the HSV-1 helicase–primase complex [34]. HSV-1, like ASFV, is a dsDNA virus whose core replication machinery includes the UL5 helicase, which shares conserved ATPase motifs (Walker A/B) and serves as the direct binding target of Pritelivir [33]. Thus, the efficacy of Pritelivir against ASFV represents a cross-viral target validation, likely predicated on structural conservation within the helicase core domains of HSV-1 UL5 and ASFV A859L. Treatment with Pritelivir resulted in a concentration-dependent reduction in intracellular viral proteins (p30, p72), mRNA levels, and extracellular infectious virions without inducing cytotoxicity (Figure 6). Although direct biochemical validation of Pritelivir binding to A859L—such as resistance mutation mapping or in vitro enzymatic assays—remains to be performed, multiple lines of evidence support its on-target specificity. Notably, RT-qPCR analysis at early time points post-infection (9 hpi) confirmed that *A859L* mRNA levels were unaltered by inhibitor treatment (Figure S2). This indicates that Pritelivir impairs A859L function at the protein level rather than suppressing gene transcription, thereby arguing against nonspecific transcriptional toxicity or off-target effects. Collectively, these pharmacological data, combined with our structural and functional findings, strongly suggest that A859L helicase activity is mechanistically crucial for efficient ASFV replication.

Our pharmacological inhibition experiments were conducted in the context of wild-type virus infection, where no compensatory genetic adaptations exist, thereby revealing the acute dependence of viral replication on A859L enzymatic function. It is noteworthy that both siRNA-mediated knockdown (Figure 4 and Figure 5) and pharmacological inhibition (Figure 6) attenuated, but did not completely abolish, viral replication. This partial phenotype suggests that while A859L is critical for efficient viral gene expression, it may not represent the sole rate-limiting factor in the ASFV replication cycle; residual replication could be sustained by redundant or compensatory mechanisms within the viral replication–transcription complex. Nevertheless, this observation aligns with the broader principle that targeting critical viral enzymes such as helicases is a viable antiviral strategy. Previous studies have identified other potential ASFV drug targets, including the DNA repair enzyme OGG1 [43] and natural compounds such as aloe-emodin and theaflavin that act through distinct mechanisms [44,45]. Our study adds A859L to this growing list of validated targets. We hypothesize that Pritelivir inhibits the unwinding activity of A859L, thereby blocking viral gene transcription and translation and ultimately curtailing viral propagation. This work provides a solid theoretical foundation for elucidating novel pathogenic mechanisms of ASFV and for developing new antiviral strategies targeting A859L.

Collectively, these findings demonstrate that A859L plays a vital role in facilitating ASFV replication and that pharmacological inhibition of its helicase activity by Pritelivir effectively suppresses viral propagation. These data establish A859L as a critical determinant of ASFV replicative fitness and a promising target for antiviral intervention.

## 5. Conclusions

Our results identify A859L as a key factor in ASFV replication. Specifically, siRNA-mediated knockdown of *A859L* significantly reduced intracellular viral mRNA and protein levels—including those of other ASFV RNA helicases—and decreased extracellular infectious virion production, underscoring its vital role in viral propagation. Conversely, A859L overexpression enhanced viral replication. Furthermore, given that A859L functions as an RNA helicase, pharmacological inhibition of its helicase activity with Pritelivir effectively suppressed viral growth. Collectively, these data establish A859L as a critical determinant of ASFV replication and a promising candidate for antiviral development.

## Figures and Tables

**Figure 1:**
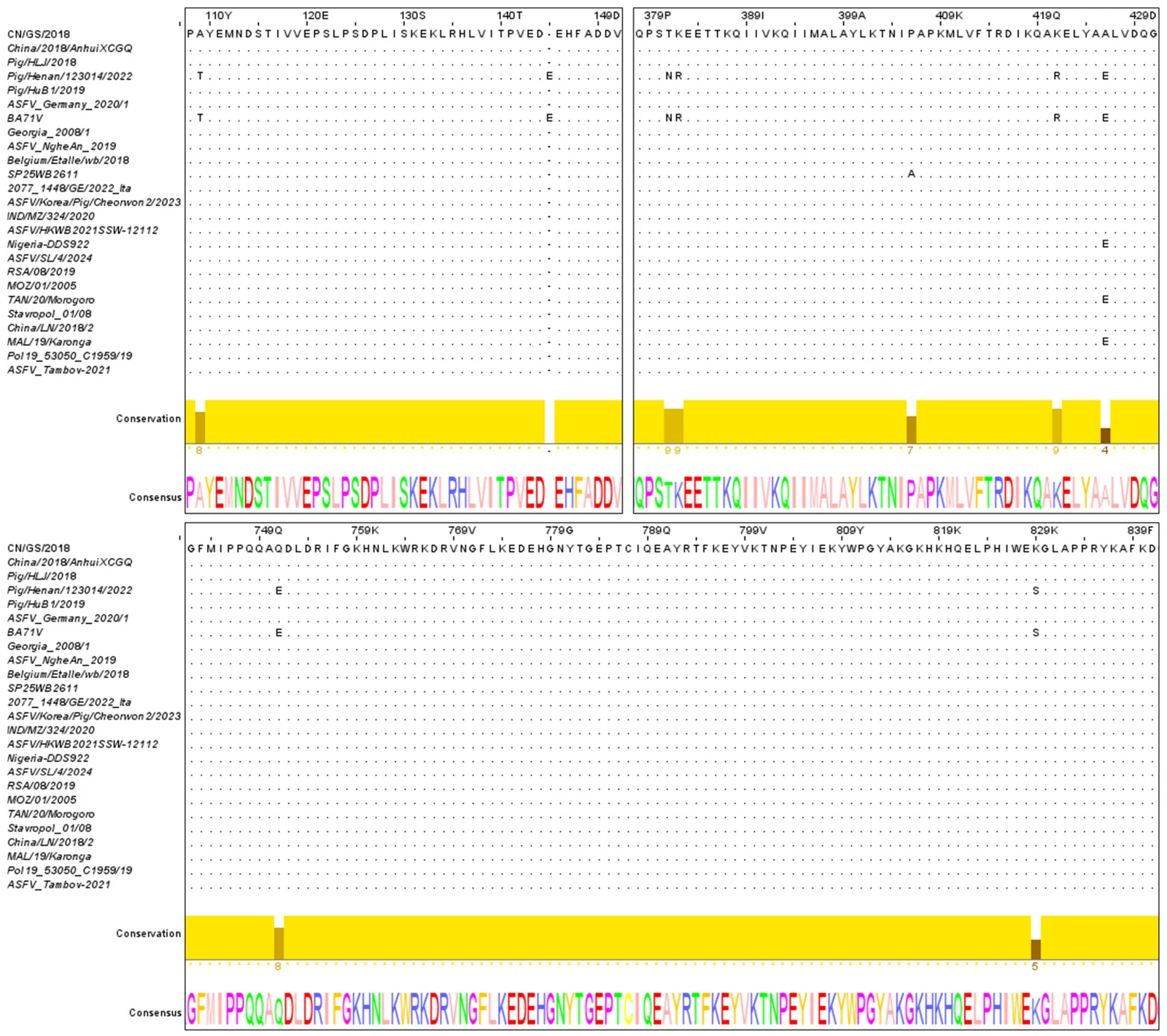
Multiple sequence alignment of A859L amino acid residues among ASFV isolates in nature. Amino acid sequences of the A859L protein from 25 representative ASFV isolates, retrieved from the NCBI Virus database, were aligned with the A859L sequence of the ASFV CN/GS/2018 strain using Jalview software (version 2.11.5.0) with the ClustalW alignment algorithm. The degree of sequence conservation across the alignment is shown below the sequences.

**Figure 2:**
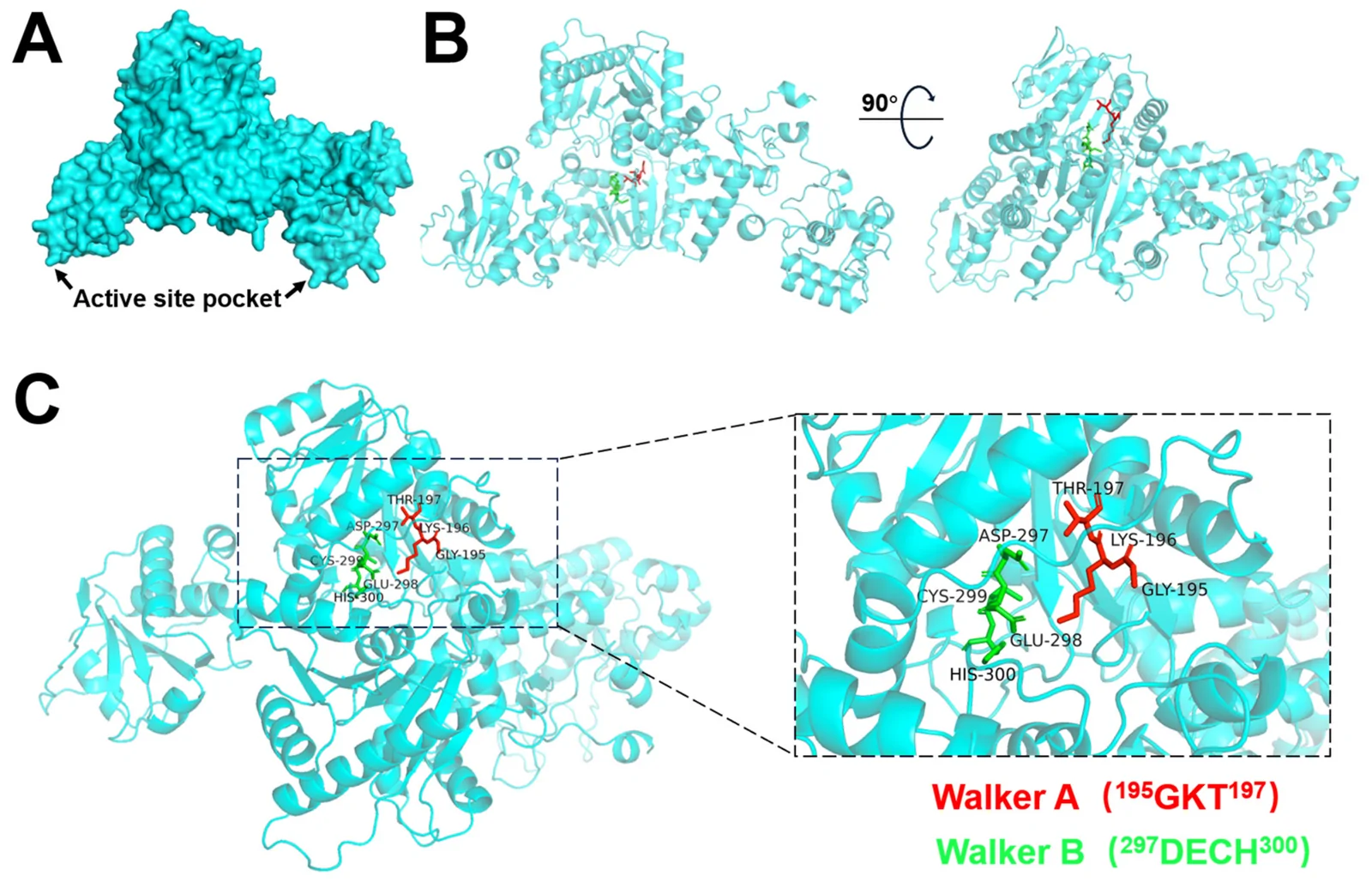
Structural characterization of the helicase active site in A859L. (**A**) The A859L protein structure was predicted via the AlphaFold 3 online platform and visualized in 3D using PyMol. Surface representation of the full-length A859L protein, revealing the overall molecular shape and surface features. (**B**) Ribbon diagram of the A859L structure in two orientations (rotated 90°), displaying the α-helical and β-sheet secondary structure elements. The Walker A (red) and Walker B (green) motifs of the helicase active site in A859L are highlighted. (**C**) Detailed ribbon view of the A859L protein with a magnified inset showing the spatial arrangement of key residues in the Walker A motif (red: Gly195, Lys196, Thr197) and Walker B motif (green: Asp297, Glu298, Cys299, His300).

**Figure 3:**
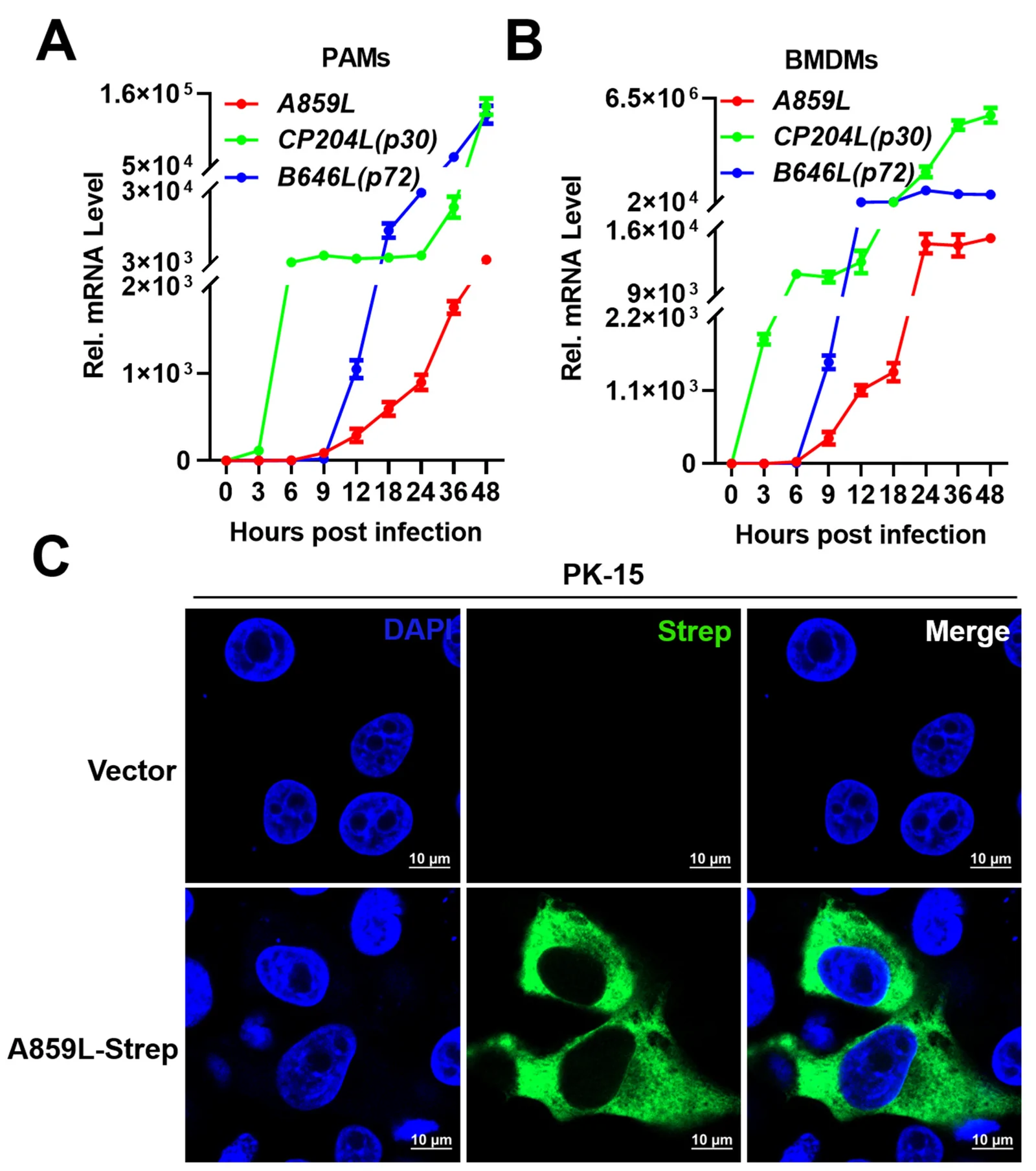
Transcriptional dynamics and intracellular localization of A859L. (**A**,**B**) RT-qPCR analysis of *A859L*, *CP204L*, and *B646L* mRNA levels in ASFV-infected PAMs or BMDMs. PAMs (**A**) or BMDMs (**B**) were infected with ASFV at MOIs of 0.5 or 0.1 and harvested at the indicated time points. Total RNA was extracted, and mRNA expression levels of *A859L*, *CP204L*, and *B646L* were quantified by RT-qPCR. *GAPDH* served as the internal reference gene for normalization. (**C**) Intracellular localization of A859L in PK-15 cells assessed by confocal microscopy. PK-15 cells were transfected with the pRK-A859L-Strep plasmid expressing A859L-Strep for 24 h, fixed, and immunostained with an anti-Strep antibody (green) to detect A859L. Nuclei were counterstained with DAPI (blue). Scale bars: 10 μm. Data represent the mean ± SD from the three independent experiments.

**Figure 4:**
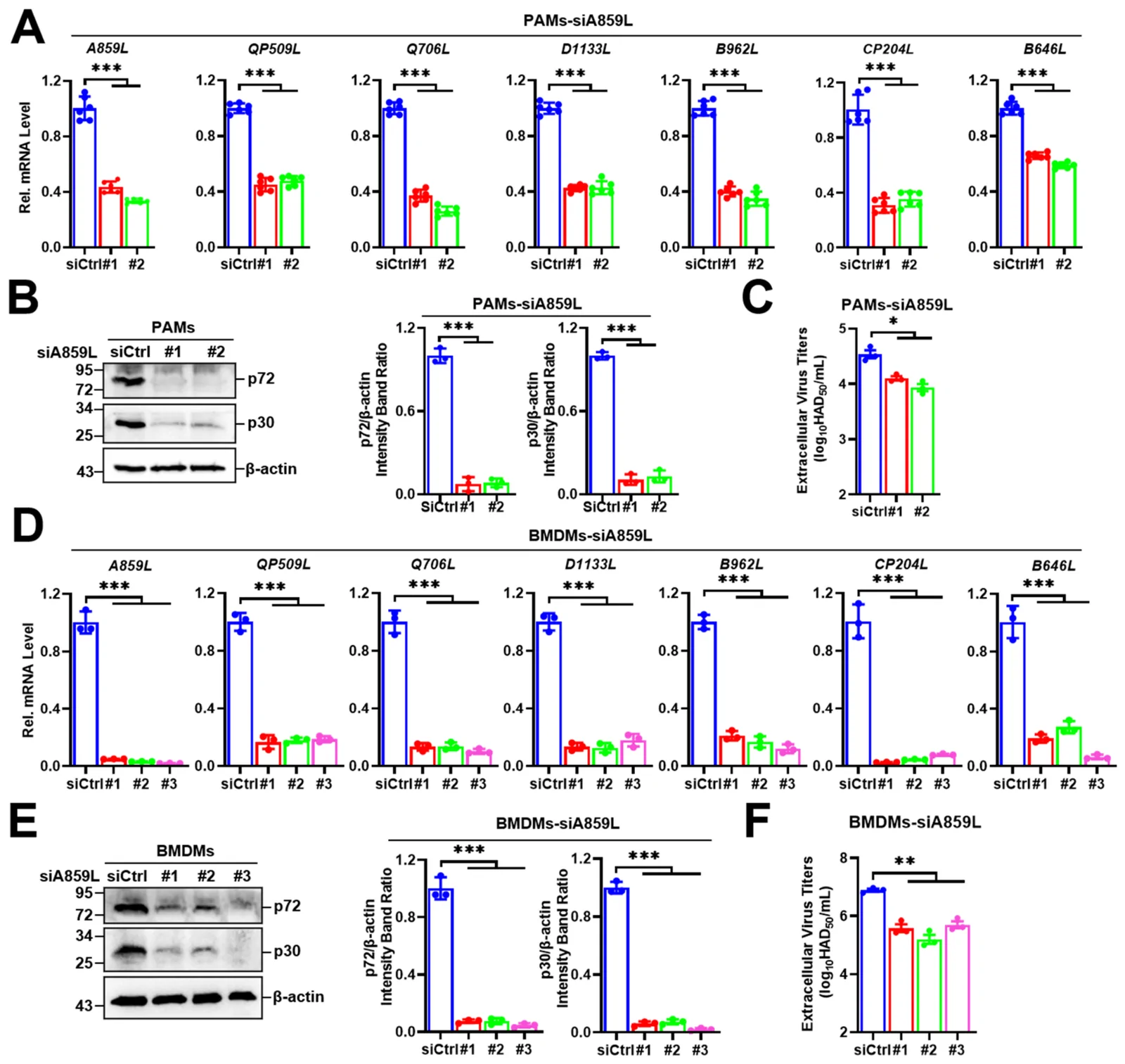
A859L is crucial for ASFV replication. (**A**) Effect of *A859L* knockdown on the expression of other helicase genes in ASFV-infected PAMs. PAMs were transfected with control siRNA (siCtrl) or one of two *A859L*-specific siRNAs (siA859L#1 and #2). At 24 h post-transfection (hpt), cells were infected with ASFV (MOI = 0.5). Total RNA was extracted at 36 hpi and the mRNA levels of *A859L*, *QP509L*, *Q706L*, *D1133L*, *B962L*, *CP204L* and *B646L* were quantified by RT-qPCR. *GAPDH* served as the internal reference gene for normalization. (**B**) Western blot analysis of viral proteins p72 and p30 in ASFV-infected *A859L*-knockdown PAMs is described in panel A. p72 and p30 were detected by the anti-p72 antibody and anti-p30 antibody, respectively. Relative band intensities of p72 and p30 from three independent experiments were quantified using Image J and normalized to β-actin (right). (**C**) Extracellular viral titers in the supernatants from panel B were determined by hemadsorption assay. (**D**) Effect of *A859L* knockdown on the expression of other helicase genes in ASFV-infected BMDMs. BMDMs were transfected with siCtrl or one of two *A859L*-specific siRNAs. At 24 hpt, cells were infected with ASFV (MOI = 0.1). Total RNA was extracted at 36 hpi and the mRNA levels of *A859L*, *QP509L*, *Q706L*, *D1133L*, *B962L*, *CP204L* and *B646L* were quantified by RT-qPCR. (**E**) Western blot analysis of p72 and p30 in ASFV-infected *A859L*-knockdown BMDMs. Relative band intensities are shown in the right panel. (**F**) Extracellular viral titers in the supernatants from panel E were measured by hemadsorption assay. Data represent the mean ± SD from at least three independent experiments. Statistical significance was determined by one-way ANOVA (* *p* < 0.05; ** *p* < 0.01; *** *p* < 0.001).

**Figure 5:**
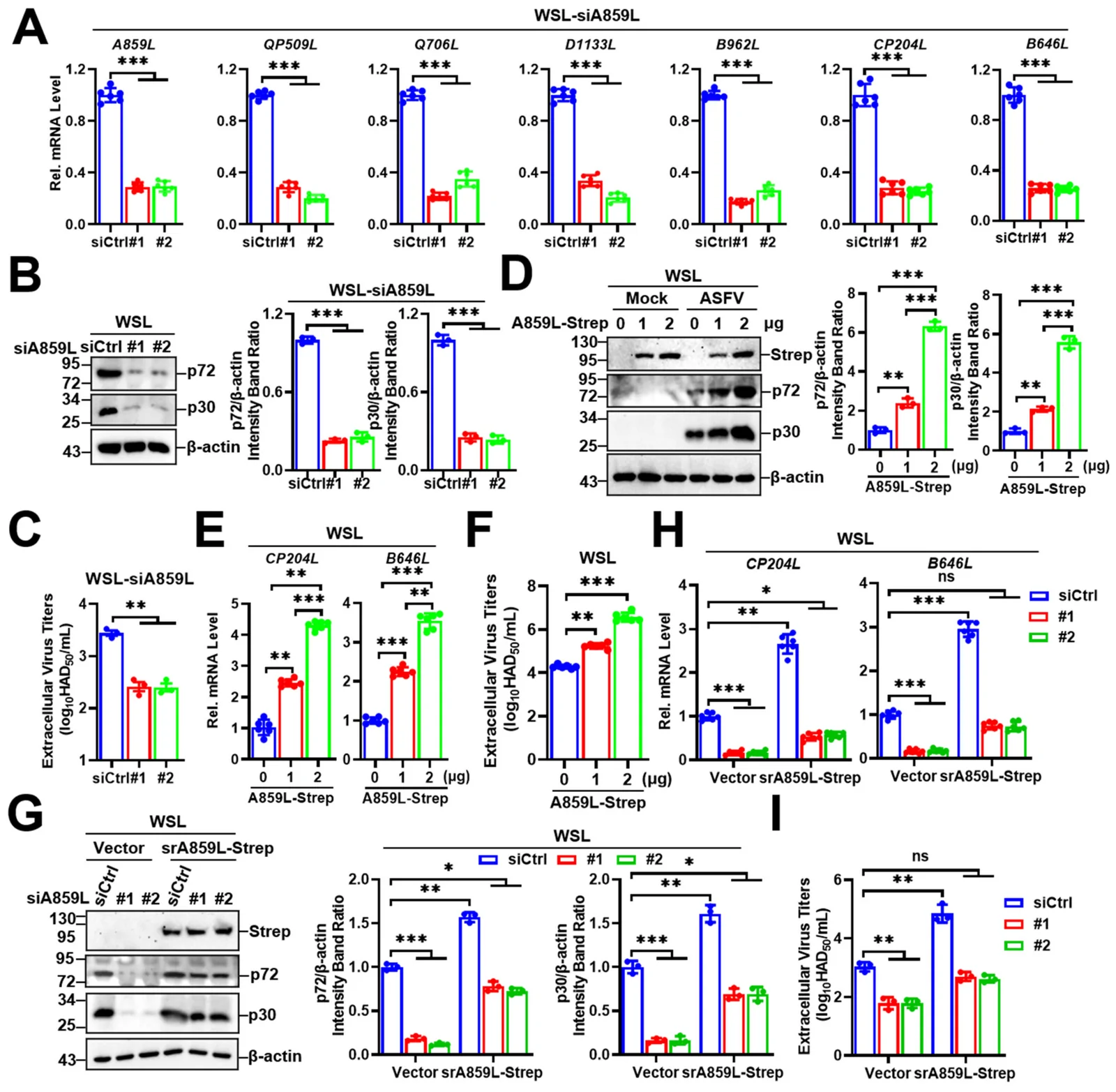
A859L promotes ASFV replication. (**A**) Effect of *A859L* knockdown on the expression of other helicase genes in ASFV-infected WSL cells. Cells were transfected with siCtrl or one of two *A859L*-specific siRNAs (siA859L#1 and #2). At 18 hpt, cells were infected with ASFV (MOI = 2). Total RNA was extracted at 36 hpi, and mRNA levels of *A859L*, *QP509L*, *Q706L*, *D1133L*, *B962L*, *CP204L*, and *B646L* were quantified by RT-qPCR. *GAPDH* served as the internal reference for normalization. (**B**) Western blot analysis of viral proteins p72 and p30 in ASFV-infected *A859L*-knockdown WSL cells is described in panel A. Band intensities of p72 and p30 were quantified and normalized (right). (**C**) Extracellular viral titers in supernatants from the experiment in panel B were determined by hemadsorption assay. (**D**) Western blot analysis of the effect of A859L overexpression on ASFV replication in WSL cells. Cells were transfected with an empty vector (2 μg/well) or increasing amounts of a A859L-Strep expression plasmid (1 or 2 μg/well). At 24 hpt, cells were mock-infected or infected with ASFV (MOI = 2) for 36 h. Lysates were analyzed by Western blot using anti-Strep, anti-p72, and anti-p30 antibodies (left). Relative band intensities of p72 and p30 were quantified and normalized to β-actin (right). (**E**) RT-qPCR analysis of *CP204L* and *B646L* mRNA levels in the ASFV-infected WSL cells described in panel (**D**). (**F**) Extracellular viral titers in supernatants from the experiment in panel (**D**) were determined by hemadsorption assay. (**G**) Rescue of viral replication by siRNA-resistant, Strep-tagged A859L in *A859L*-knockdown WSL cells. Cells were transfected with either siCtrl or one of two *A859L*-specific siRNAs for 18 h, infected with ASFV for 12 h, and subsequently transfected with an empty vector (1 μg/well) or a plasmid expressing siRNA-resistant, Strep-tagged A859L (pRK-srA859L-Strep, 1 μg/well). Cells were harvested at 36 hpi and subjected to Western blot analysis using anti-Strep, anti-p72, and anti-p30 antibodies. Band intensities of p72 and p30 were quantified (right). (**H**) RT-qPCR analysis of *CP204L* and *B646L* mRNA levels in the ASFV-infected WSL cells described in panel (**G**). (**I**) Extracellular viral titers in supernatants from the experiment in panel (**G**) were determined by hemadsorption assay. Data represent the mean ± SD from three independent experiments. Statistical significance was determined by one-way ANOVA (* *p* < 0.05; ** *p* < 0.01; *** *p* < 0.001).

**Figure 6:**
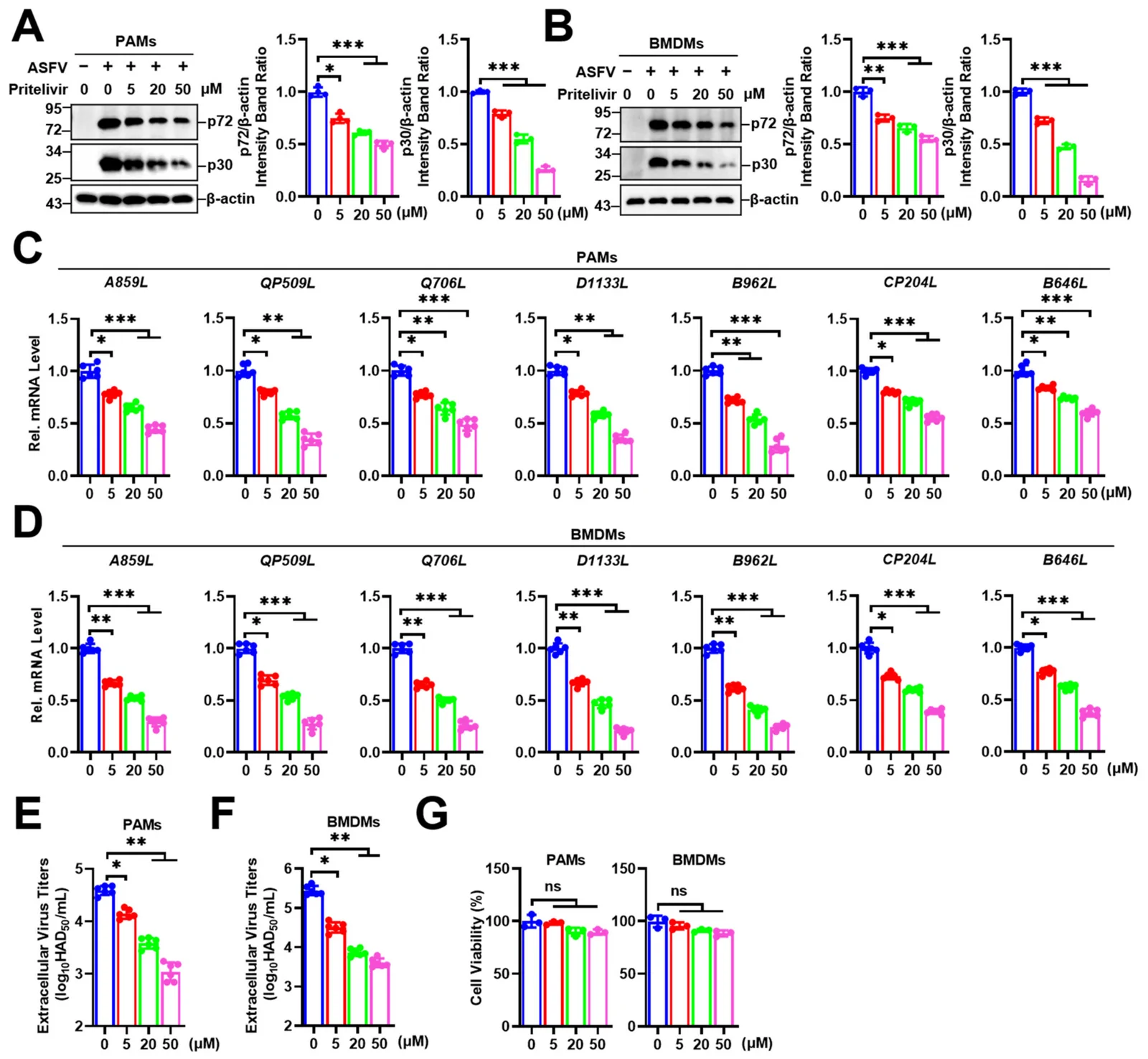
The helicase activity is critical for ASFV replication. (**A**,**B**) The effect of the helicase inhibitor Pritelivir on ASFV replication was assessed by Western blot. PAMs (**A**) and BMDMs (**B**) were mock-infected or infected with ASFV (MOI = 0.5 or 0.1) for 2 h, followed by incubation with Pritelivir at the indicated concentrations for an additional 36 h. Cell lysates were harvested and analyzed. The relative gray values of the p72 and p30 bands were quantified (right panels). (**C**,**D**) Effect of the helicase inhibitor Pritelivir on the expression of ASFV helicase genes. Cells from ASFV-infected *A859L*-knockdown PAMs ((**C**); corresponding to panel (**A**)) and BMDMs ((**D**); corresponding to panel (**B**)) were harvested for total RNA extraction. The mRNA levels of *A859L*, *QP509L*, *Q706L*, *D1133L*, *B962L*, *CP204L* and *B646L* were quantified by RT-qPCR. (**E**,**F**) Extracellular viral titers in the supernatants from panel (**A**) (PAMs, (**E**)) and panel (**B**) (BMDMs, (**F**)) were determined by hemadsorption assay. (**G**) Cytotoxicity of Pritelivir in PAMs and BMDMs was evaluated using a CCK-8 assay. Cells were cultured with the indicated concentrations of Pritelivir for 36 h, and viability was measured relative to the DMSO control (0 μM). Data represent the mean ± SD from three independent experiments. Statistical significance was determined by one-way ANOVA (* *p* < 0.05; ** *p* < 0.01; *** *p* < 0.001; Not significant (ns): *p* > 0.05).

**Table 1:** Primers used for RT-qPCR.

Genes	Forward Sequences (‘5-3’)	Reverse Sequences (‘5-3’)
*GAPDH*	ACATGGCCTCCAAGGAGTAAGA	GATCGAGTTGGGGCTGTGACT
*A859L*	CTTGGAAACTTGGCTTGCCC	AACAGTGGTTAGGACGCCTG
*QP509L*	TACGGGCGTAGAGGCATTTC	GATCCCTGCCGGGTAATAC
*Q706L*	AAAGAGCGCGACATGATCCA	GCCTTATCCCAGTACGGCTC
*D1133L*	GATTCGCGATGAGCACACAC	CACAATCACCTTGGACCCGA
*B962L*	ACATTAAGACCGCCTGCGAA	AGCCATACCGGGCATGAAAA
*CP204L*	GAGGAGACGGAATCCTCAGC	TAGGTACCTCCGATGAGGGC
*B646L*	GCGCTCTGGATTAAGTTGCG	ATATTGCGTCTACTGGGGCG

**Table 2:** siRNA sequences used in this study.

Names	Forward Sequences (‘5-3’)	Reverse Sequences (‘5-3’)
Control siRNA	UUCUCCGAACGUGUCACGUTT	ACGUGACACGUUCGGAGAATT
si*A859L*#1	CGAAACAGAUCAUAGUAAAGC	UUUACUAUGAUCUGUUUCGUG
si*A859L*#2	GAUGAGACACGGACUGCAAUC	UUGCAGUCCGUGUCUCAUCUG
si*A859L*#3	UAAUUCUUGGUGUUUAUGCUU	GCAUAAACACCAAGAAUUACC

## Data Availability

All data are contained within the article.

## Supplementary Materials

The following supporting information can be downloaded at: https://www.mdpi.com/article/10.3390/v18101059/s1, Figure S1: Multiple sequence alignment of A859L amino acid residues among ASFV isolates in nature; Figure S2: Inhibition of helicase activity does not affect ASFV helicases gene expression at early time points post-infection.
