# Supplementary material for: A859L Plays a Critical Role in Facilitating Efficient African Swine Fever Virus Replication
Source: Viruses. 2026 Sep 24;18(10):1059. doi: 10.3390/v18101059 (PMC13709718; doi:10.3390/v18101059)
Supplement: Supplementary file 1 [file viruses-18-01059-s001.zip › Supplemental legends.pdf]

# SUPPLEMENTAL MATERIAL

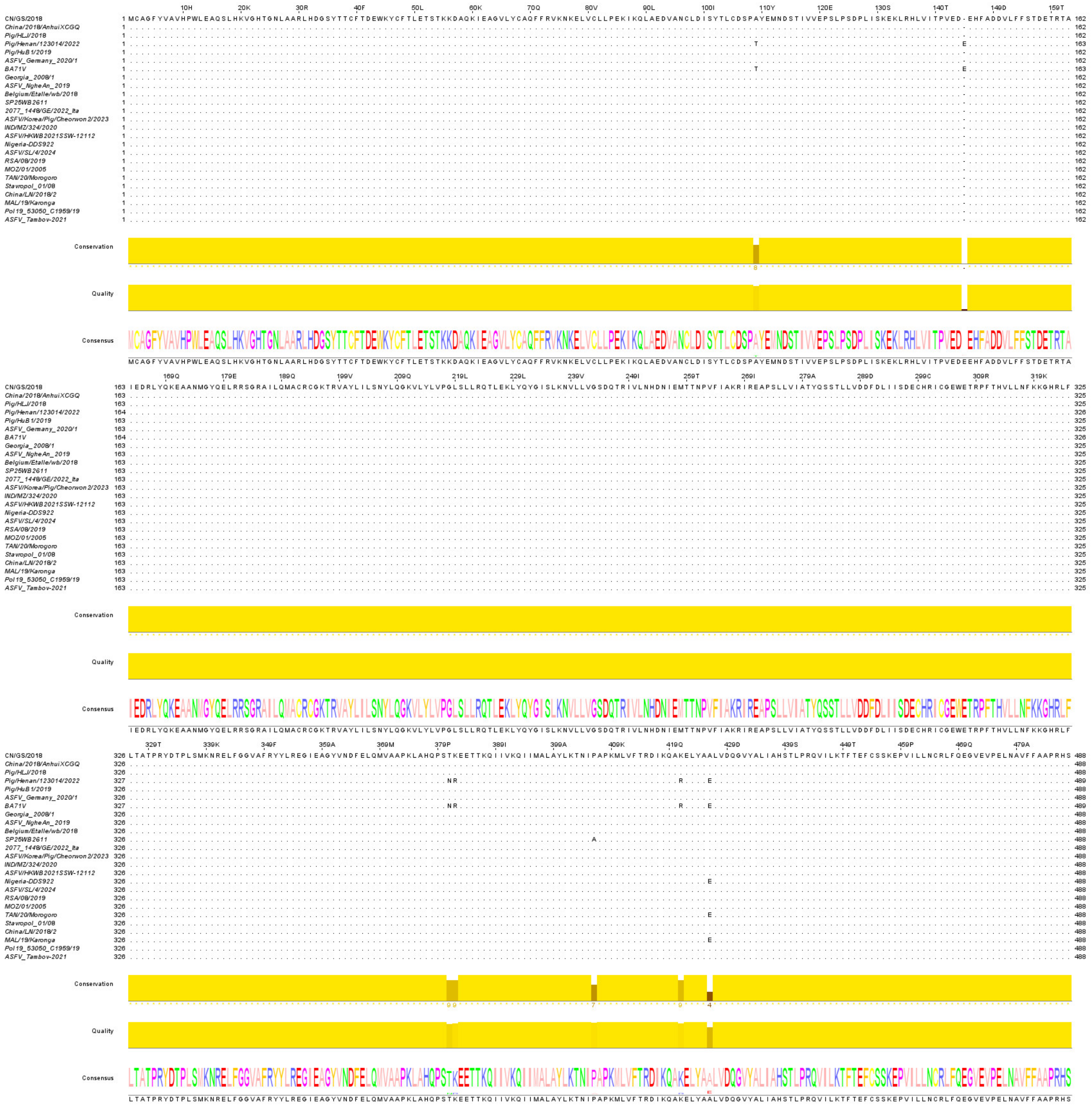

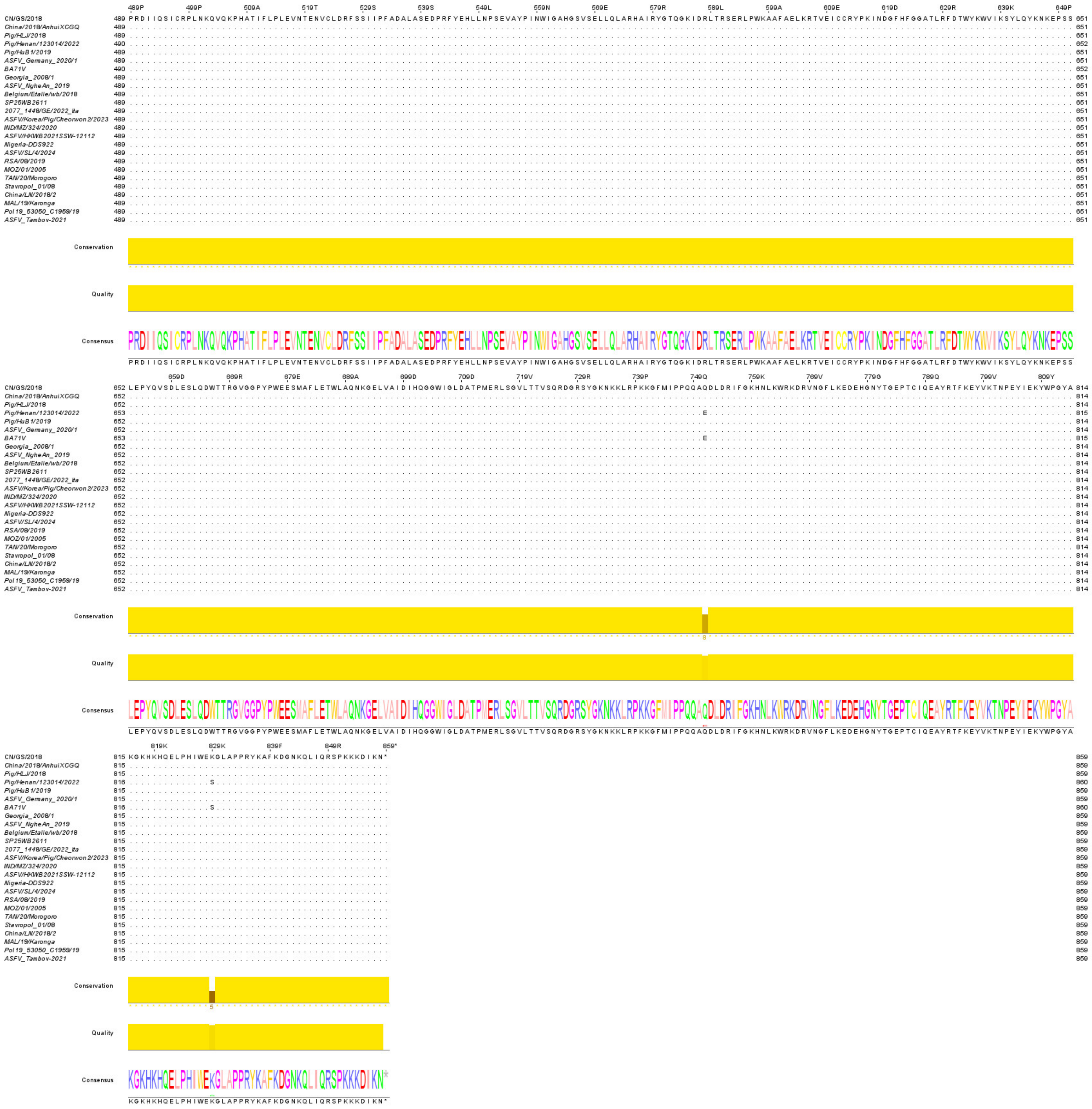


**Figure S1.** Multiple sequence alignment of A859L amino acid residues among ASFV isolates in nature. Amino acid sequences of the A859L protein from 25 representative ASFV isolates, retrieved from the NCBI Virus database, were aligned with the pA859L sequence of the ASFV CN/GS/2018 strain using Jalview software (version 2.11.5.0) with the ClustalW alignment algorithm. The degree of sequence conservation across the alignment is shown below the sequences.

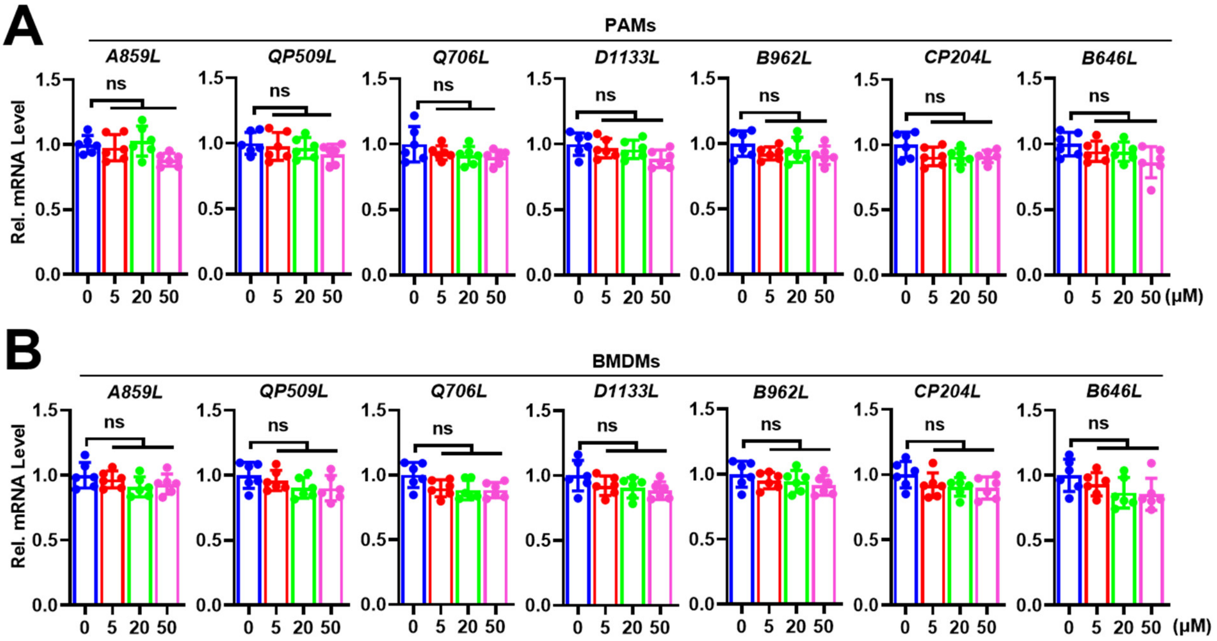


**Figure S2.** Inhibition of helicase activity does not affect ASFV helicases gene expression at early time points post-infection. (**A and B**) PAMs (A) and BMDMs (B) were infected with ASFV at an MOI of 0.5 or 0.1, respectively, for 2 h, followed by treatment with Pritelivir at the indicated concentrations for an additional 7 h. Total RNA was extracted, and mRNA expression levels of *A859L*, *QP509L*、*Q706L*、*D1133L*、*B962L*、*CP204L*, and *B646L* were quantified by RT-qPCR. *GAPDH* served as the internal reference gene for normalization.
